# Phosphorylation of GntR reduces *Streptococcus suis* oxidative stress resistance and virulence by inhibiting NADH oxidase transcription

**DOI:** 10.1371/journal.ppat.1011227

**Published:** 2023-03-13

**Authors:** Kai Niu, Yu Meng, Mingxing Liu, Zhe Ma, Huixing Lin, Hong Zhou, Hongjie Fan

**Affiliations:** 1 MOE Joint International Research Laboratory of Animal Health and Food Safety, College of Veterinary Medicine, Nanjing Agricultural University, Nanjing, China; 2 Jiangsu Co-innovation Center for Prevention and Control of Important Animal Infectious Diseases and Zoonoses, Yangzhou University, Yangzhou, China; The University of Alabama at Birmingham, UNITED STATES

## Abstract

GntR transcription factor of *Streptococcus suis* serotype 2 (SS2) is a potential substrate protein of STK, but the regulation mechanisms of GntR phosphorylation are still unclear. This study confirmed that STK phosphorylated GntR *in vivo*, and *in vitro* phosphorylation experiments showed that STK phosphorylated GntR at Ser-41. The phosphomimetic strain (GntR-S41E) had significantly reduced lethality in mice and reduced bacterial load in the blood, lung, liver, spleen, and brain of infected mice compared to wild-type (WT) SS2. Electrophoretic mobility shift assay (EMSA) and chromatin immunoprecipitation (ChIP) experiments demonstrated that the promoter of *nox* was bound by GntR. The phosphomimetic protein GntR-S41E cannot bind to the promoter of *nox*, and the *nox* transcription levels were significantly reduced in the GntR-S41E mutant compared to WT SS2. The virulence in mice and the ability to resist oxidative stress of the GntR-S41E strain were restored by complementing transcript levels of *nox*. NOX is an NADH oxidase that catalyzes the oxidation of NADH to NAD^+^ with the reduction of oxygen to water. We found that NADH is likely accumulated under oxidative stress in the GntR-S41E strain, and higher NADH levels resulted in increased amplified ROS killing. In total, we report GntR phosphorylation could inhibit the transcription of *nox*, which impaired the ability of SS2 to resist oxidative stress and virulence.

## Author summary

Serine/threonine kinase (STK) of SS2 is involved in various important biological processes, and is crucial to bacteria pathogenicity. Our work here shows that STK can inhibit the transcription of the *nox* gene by phosphorylating the transcription factor GntR, which weakens the anti-oxidant capacity and virulence. SS2 lacking NOX is more likely to produce the accumulation of NADH under oxidative stimulation, which increases amplified ROS killing.

## Introduction

*Streptococcus suis* serotype 2 (SS2) is a major bacterial pathogen that causes meningitis, septicemia, and arthritis in pigs and serious economic losses in the swine industry [1,2]. Humans can be infected through close contact with infected pigs or their products, mainly with meningitis and streptococcal toxic shock-like syndrome (STSLS) [3]. *Streptococcus suis* has caused thousands of cases of human disease in China, Vietnam, and Thailand and has been identified as one of the most common forms of bacterial meningitis in adults [4,5].

The GntR family of transcription factors is widely distributed in bacteria and can be involved in the control of various metabolic processes. They usually contain two domains: an N-terminal DNA-binding domain and a C-terminal effector-binding domain [6]. GntR family transcription factors are divided into 7 subfamilies including FadR, HutC, MocR, YtrA, AraR, DevA, and PlmA, based on the similarity with effector binding domains [7–9]. They are involved in capsular polysaccharide synthesis and virulence in *Streptococcus pneumoniae*, expression of sugar transporter, biofilm synthesis, and antibiotic resistance in *Streptococcus mutans* [10–12]. STKs have been found to achieve phosphorylation regulation in a variety of ways [13–15]. However, the impact of STK phosphorylation modification of GntR on its regulatory function is still unknown.

Reactive oxygen species (ROS) produced by host phagocytes can clear pathogens and is an important host defense mechanism. Therefore, bacteria’s ability to resist oxidative stress is a virulence-related property [16]. Bacteria have developed a variety of anti-oxidant mechanisms to deal with the damage caused by oxidation [17]. These anti-oxidant systems work in concert to protect proteins, DNA, and lipids of cells from oxidative damage [18]. Nicotinamide adenine dinucleotide hydride oxidase (NADH Oxidase, NOX) regulates oxidative stress, substance transport, and energy metabolism by metabolizing oxygen and regulating the ratio of NADH/NAD^+^, which is essential for bacterial growth and virulence [19]. The reoxidation of NADH to NAD^+^, accompanied by the reduction of oxygen by the electron transport chain, and provides the main source of ATP for aerobic organisms. The NADH/NAD^+^ ratio is considered to be a sensitive indicator of the redox state because NADH levels are increased when oxygen is unavailable or limiting or the electron transport chain is compromised [20]. In *mycobacteria*, increased NADH concentrations can mediate bacterial resistance to isoniazid and ethionamide [21]. But the relationship between NOX, NADH/NAD^+^ ratio and resistance in SS2 is unclear.

In this study, we used immunoprecipitation to demonstrate that GntR is phosphorylated *in vivo* and determined that STK phosphorylates GntR at S41 by a combination of mass spectrometry, and targeted mutagenesis. Phosphorylation of GntR S41 represses GntR binding to the *nox* promoter and significantly affects oxidative stress tolerance and virulence in SS2.

## Results

### GntR is phosphorylated by STK

**Fig 1:**
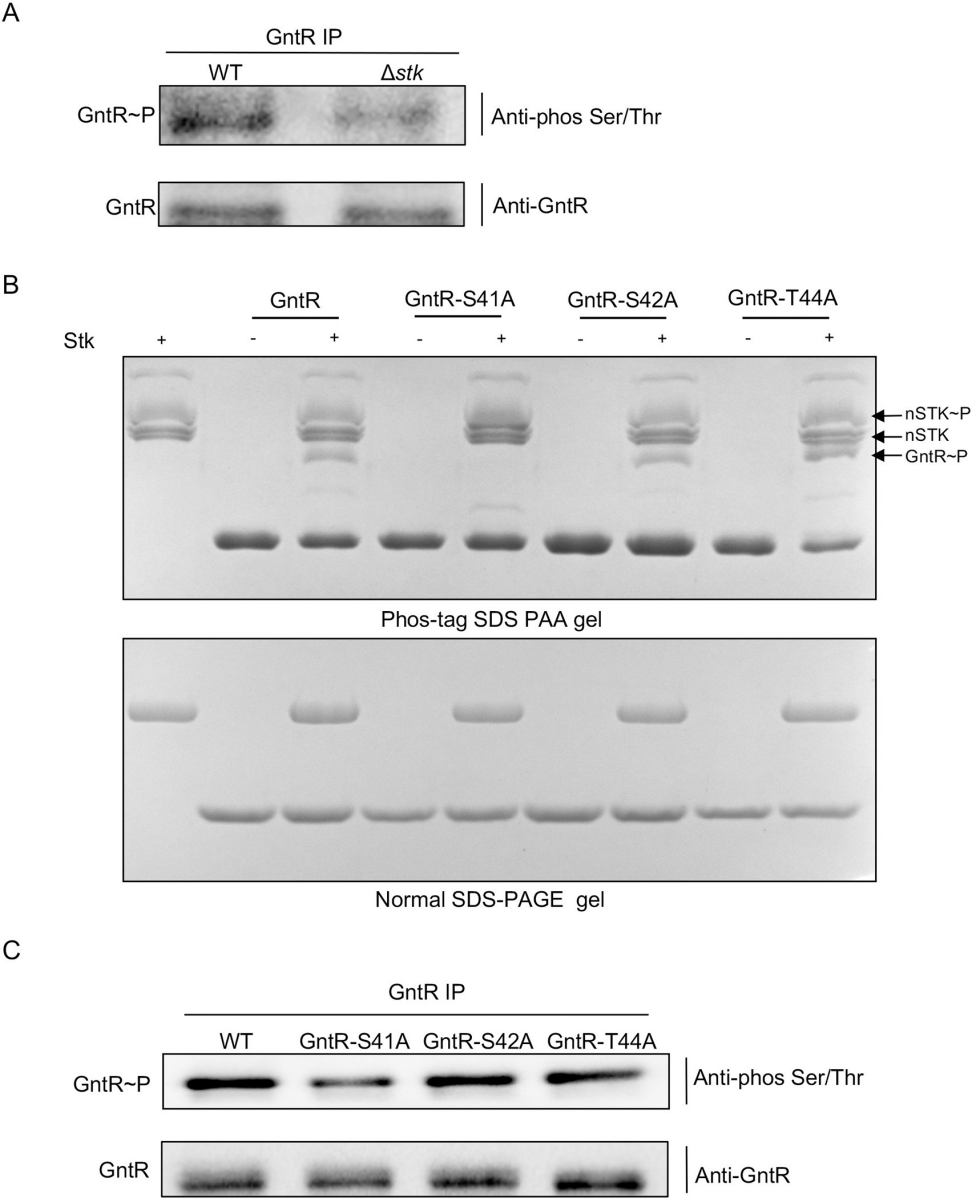
GntR is phosphorylated by STK. (A) GntR polyclonal antibody was used to perform immunoprecipitation (IP) experiments with WT SS2 and Δ*stk* whole proteins, respectively. The pull-down protein was separated by SDS-PAGE, and Western blot analysis was performed using GntR polyclonal antibody and Anti-phos Ser/Thr antibody at 1:1000 dilution, respectively. (B) Phosphorylation reactions were carried out with purified GST-nSTK (15 μg), and substrate proteins (5 μg) mixed in phosphorylation buffer and incubated at 37°C for 2 h. Samples were loaded on 10% Phosbind Acrylamide SDS-PAGE (top) and conventional SDS-PAGE (bottom). Separated proteins were visualized by Coomassie Brilliant Blue staining. (C) GntR polyclonal antibody was used to perform immunoprecipitation (IP) experiments with WT SS2, GntR-S41A, GntR-S42A, and GntR-T44A whole proteins, respectively. The pull-down protein was detected using the method in (A).

According to the results of phosphoproteomics, a phospho-peptide signal of GntR (ZY05719_02215) in Δ*stk* was significantly attenuated compared with WT SS2 (S1 Table), and the mass spectrometry results showed that Ser-42 was a potential phosphorylation site (S1 Fig). To verify whether STK could affect the phosphorylation level of GntR in *vivo*, immunoprecipitation assays were performed, which showed similar results. The phosphorylation level of GntR was significantly attenuated in Δ*stk* (Fig 1A). Next, we tried to confirm the STK-mediated phosphorylation site in GntR. Ser-42 to Ala-42 mutation was generated by PCR-mediated site-directed mutagenesis, and the recombinant GntR was expressed, purified, and incubated with nSTK *in vitro*. The phosphorylation of GntRs was examined by using the Phos-tag SDS-PAGE assay. The results showed a shifted band in both GntR and GntR-S42A proteins (Fig 1B). Analysis of the phosphoproteomics data found that the adjacent sites Ser-41 and Ter-44 were discarded because the localization probability was lower than 0.75. For mass spectrometry, it is not easy to distinguish the different phosphorylation sites, especially two adjacent sites on a peptide [22]. Therefore, we also expressed the GntR-S41A and GntR-T44A proteins, respectively, and phosphorylation assays showed that the GntR-S41A protein did not have the same migratory band on the Phos-tag gel (Fig 1B). Immunoprecipitation assays were also performed, and the phosphorylation level of GntR in GntR-S41A was significantly lower than that of the other two point mutants (Fig 1C). Thus, Ser-41 in GntR was a target site for STK phosphorylation.

### The phosphorylation of GntR attenuates SS2 virulence

The *gntR*-deleted strain Δ*gntR* was constructed by electroporation of the pSET4s-*gntR* plasmid into ZY05719 (S2A Fig), and pSET4s-*gntR*-S41A and pSET4s-*gntR-*S41E were electroporated into Δ*gntR* to construct the in situ mutant strains GntR-S41A and GntR-S41E (S2B Fig). The mutations did not affect the growth rate of SS2 (S2C Fig).

**Fig 2:**
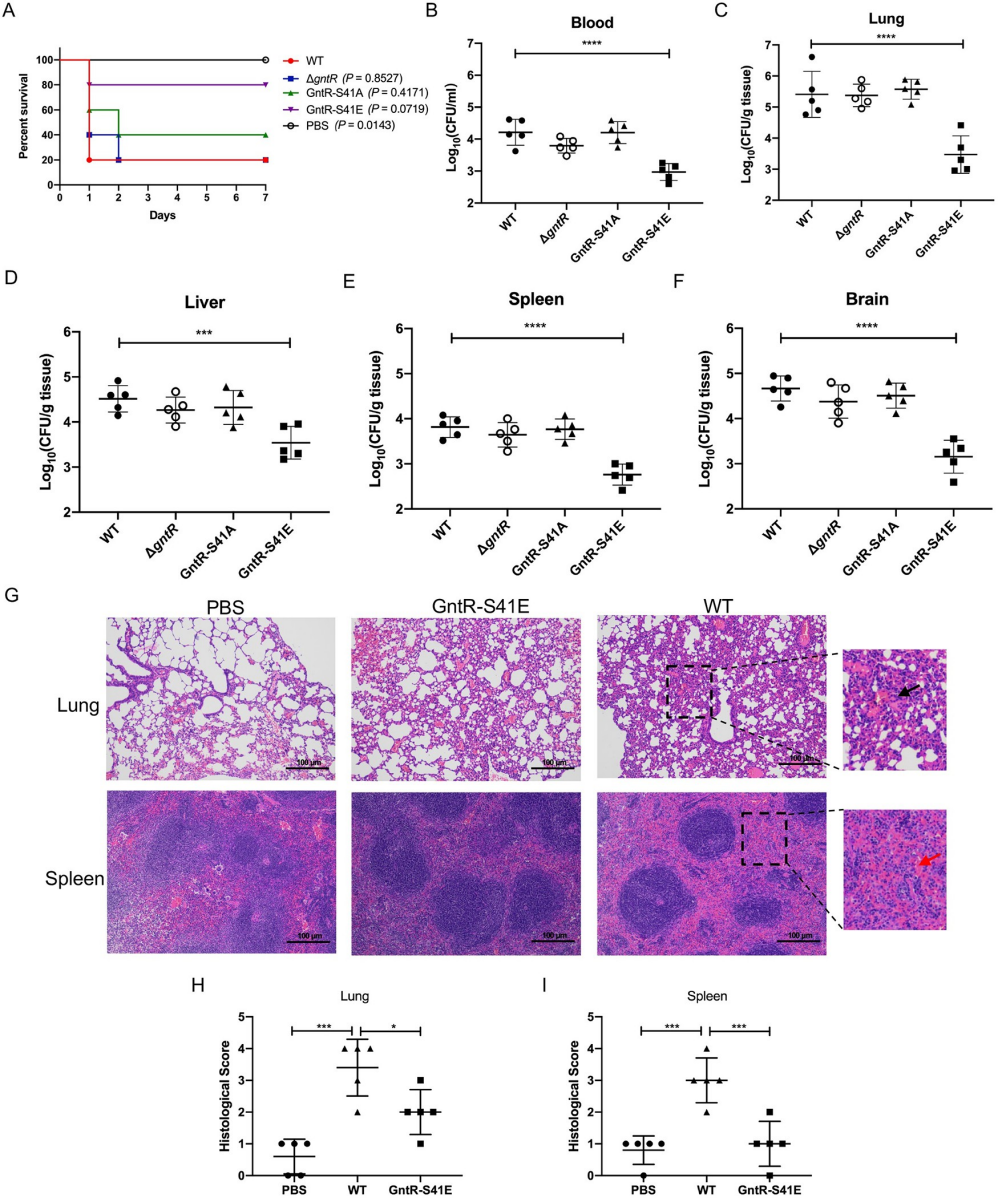
Phosphorylation of GntR reduced bacterial virulence. (A) BALB/c mice were challenged by intraperitoneal injection with WT SS2, Δ*gntR*, GntR-S41A, and GntR-S41E at a dose of 5×10^8^ CFUs/mice (n = 5 mice/group), and the survival time was monitored. (B-F) Bacterial loads in the blood (B), lung (C), liver (D), spleen (E), and brain (F) of BALB/c mice challenged with 5×10^7^ CFUs WT SS2, Δ*gntR*, GntR-S41A, and GntR-S41E by intraperitoneal injection at 24 hpi (n = 5 mice/group). (G) H&E staining of lung and spleen tissue sections from PBS, WT SS2, and GntR-S41E strain-infected mice by intraperitoneal injection at 24 hpi. In the enlarged area of the images (black dashed frame), the "black arrow" indicates alveolar congestion, collapse, and thickening. The "red arrow" indicates the decreased number of lymphocytes. Scale bar, 100 μm. (H-I) Pathological analysis of lung (H) and spleen (I) by blinded assessment of H&E stained sections. Data were statistically analyzed using Long-rank (A) and One-way ANOVA (B-F, H-I). *, *P* < 0.05; ***, *P* < 0.001; ****, *P* < 0.0001.

An animal challenge test was performed to assess the role of GntR in SS2 virulence. In this model, the mice injected with the GntR-S41E strain showed a significantly increased rate (80%) compared to mice injected with the WT SS2, which showed 20% survival (Fig 2A). To investigate the function of *gntR* in *in vivo* infection of SS2, the colonization efficiency of the WT SS2 and mutant strains in BALB/c mice was compared. At 24 h post infection (hpi), mice were sacrificed, and blood and tissue samples were collected. The CFUs in the blood, lungs, livers, spleens, and brains of mice infected with WT SS2 were significantly higher than those in the blood, lungs, livers, spleens, and brains of mice infected with GntR-S41E strain, indicating that the phosphorylation of GntR increased microbial clearance from the tissue of the infected mice (Fig 2B–2F).

Furthermore, histopathological analysis of lung and spleen tissues from mice challenged with WT SS2 revealed alveolar congestion, alveolar wall thickening, and alveolar collapse; the spleen of the infected mice developed with a decreased number of lymphocytes (Fig 2G). The lung and spleen tissues of the GntR-S41E strain-infected mice exhibited mild pathological manifestations (Fig 2G–2I). Collectively, these *in vivo* assays showed that the phosphorylation of GntR was essential in the virulence of SS2.

### Identification of genes regulated by GntR

**Fig 3:**
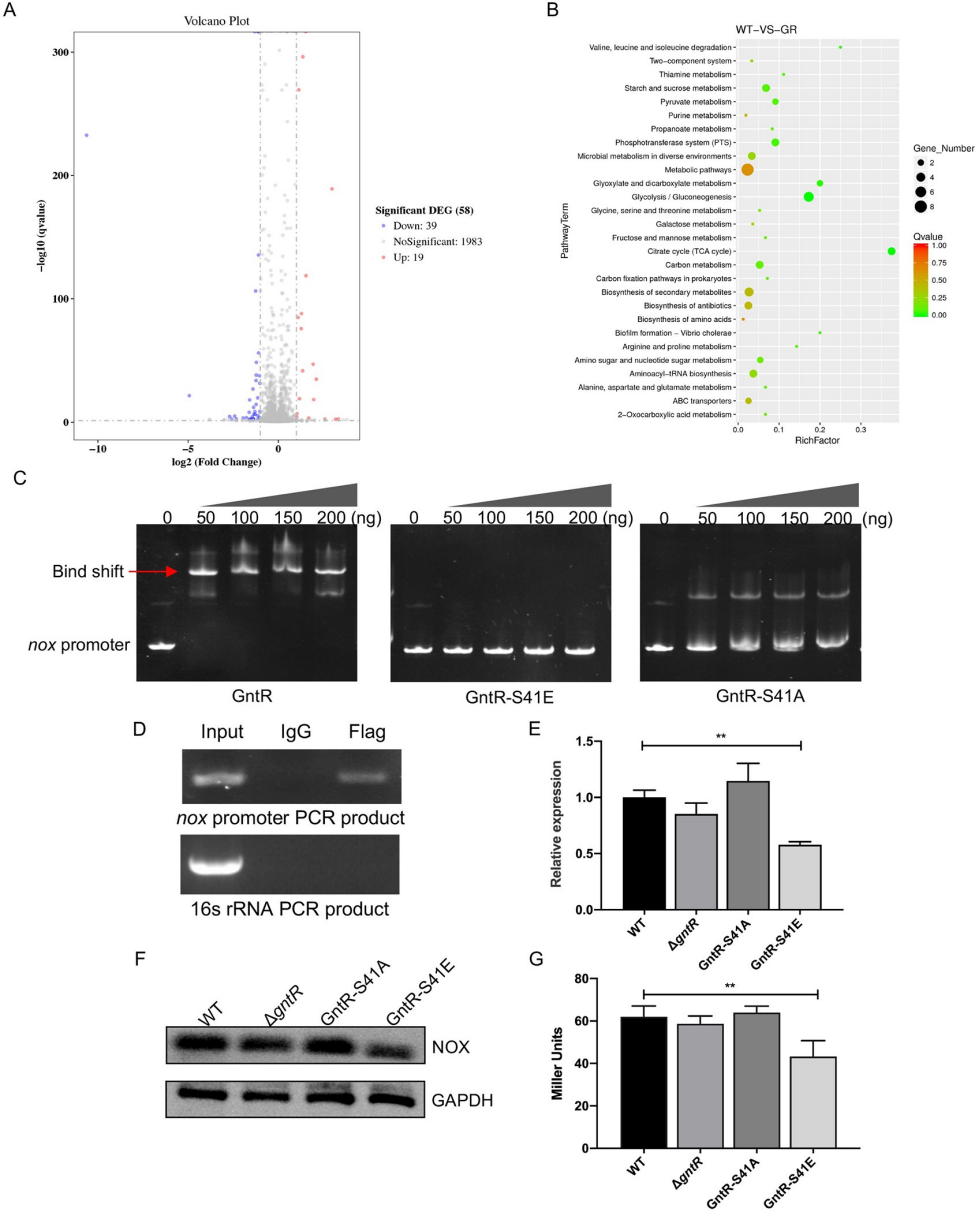
Phosphorylation of GntR inhibits transcription of *nox*. (A) Volcano plot displaying differential gene expression by RNA-seq in WT SS2 versus Δ*gntR*. The genes that are significantly up-regulated or down-regulated with at least 2-fold have been highlighted as red and blue dots, respectively. (B) KEGG pathway enrichment scatters diagram of differentially expressed genes. (C) GntR, GntR-S41A, and GntR-S41E proteins were incubated with DNA fragments (80 ng) of ~200 bp length of *nox* upstream region. The reaction was carried out at 37°C for 30 min, separated on 6% polyacrylamide gels in 0.5 × TBE buffer, and stained with ethidium bromide for imaging. (D) The binding of GntR on the *nox* promoter region was detected by ChIP assay. CΔ*gntR-flag* was grown to the log phase, and after washing with PBS, the DNA was fragmented. The anti-Flag antibody was used to collect the precipitated DNA fragments interacting with GntR, and normal mouse IgG was used as the negative control. Purified DNA was used as a template to amplify the target region of the *nox* promoter. The PCR product of the 16S rRNA gene was used as a negative control. (E) The gene of *nox* transcript levels in WT SS2, Δ*gntR*, GntR-S41A, and GntR-S41E were detected by RT-qPCR. (F) The expression of NOX in WT SS2, Δ*gntR*, GntR-S41A, and GntR-S41E was detected by Western blot. (G) The pTCV-lacZ reporter plasmid containing the *nox* promoter sequence was transformed into WT SS2, Δ*gntR*, GntR-S41A, GntR-S41E strains, and the *nox*-promoter activity was determined and normalized to internal control β-galactosidase activity. The data shown represent three independent experiments, and are presented as the mean ± standard deviations. Statistical analysis was performed using One-way ANOVA (E, G). **, *P* < 0.01.

To identify the regulated genes of GntR, we determined the gene expression profiles of Δ*gntR* and WT SS2 using RNA-seq. RT-qPCR detection found that the transcription levels of *gntR* were the highest when WT SS2 was cultured to an OD_600_ of 0.8 (S3 Fig), and we extracted the total bacterial RNA of WT SS2 and Δ*gntR* of this period and performed RNA-seq analysis. Genes with transcript level differences ≥ 2.0 and *P* value ≤ 0.05 were significantly differentially expressed. Compared to WT SS2, there were 19 up-regulated genes and 39 down-regulated genes in Δ*gntR* (Fig 3A). Up-regulated genes were mainly associated with carbon metabolism, including sugar uptake transporter (*pts*^*man/fru*^), sugar metabolizing enzymes (*pmm/pgm*), and galactose metabolizing enzymes (S2 Table). Several genes involved in stress, transport, and nuclease activity were down-regulated in Δ*gntR* (S2 Table). With the help of KEGG Pathway enrichment analysis, most of those genes were related to carbon metabolism (Fig 3B).

### GntR bond to the *nox* promoter

Among the differentially expressed genes, the gene *nox* encoding NADH oxidase attracted our attention, which is involved in oxidative stress response and virulence in *Streptococcus suis* [23]. We performed electrophoretic mobility shift assays (EMSA) using different concentrations of GntR protein (ranging from 50 ng to 200 ng) and 80 ng of ~200 bp DNA fragments of the *nox* promoter region. The results showed that the GntR protein could bind to the promoter DNA sequence of *nox*, and the protein-DNA complexes increased with the increased protein concentration (Fig 3C). To examine whether our *in vitro* observations reflect *in vivo* phenomena, we fused Flag-tag to the GntR C-terminus to obtain strain CΔ*gntR-flag*, and the expression of GntR-Flag was detected by Western blot for the Flag-tag (S4A Fig). We conclude that Flag-tag insertion did not affect GntR protein function (S4B Fig). Subsequently, ChIP assays demonstrated that GntR could also directly bind to the *nox* promoter region *in vivo* (Fig 3D).

### The phosphorylation of GntR repressed *nox* transcription

To explore the effect of the phosphorylation of GntR on its regulation, we performed EMSA to test the DNA-binding ability of the phosphomimetic protein GntR-S41E on the promoter of *nox*. The results showed that GntR-S41E protein lost its ability to bind to the promoter of *nox*, while GntR-S41A protein still has a binding capacity (Fig 3C). RT-qPCR detection showed that the transcription level of *nox* was down-regulated by about 2-fold in the GntR-S41E strain compared with WT SS2 (Fig 3E). The expression of the NOX protein in the GntR-S41E strain was significantly lower than all other strains (Fig 3F). The pTCV-lacZ plasmid was transformed into WT SS2 and *gntR* variants to analyze *nox* promoter activity *in vivo*. As expected, the promoter activity of *nox* was reduced in the GntR-S41E strain (Fig 3G). This finding indicated that the phosphorylation of GntR exerted a negative regulatory effect on NOX.

### Attenuated virulence of GntR-S41E is due to transcriptional repression of *nox*

**Fig 4:**
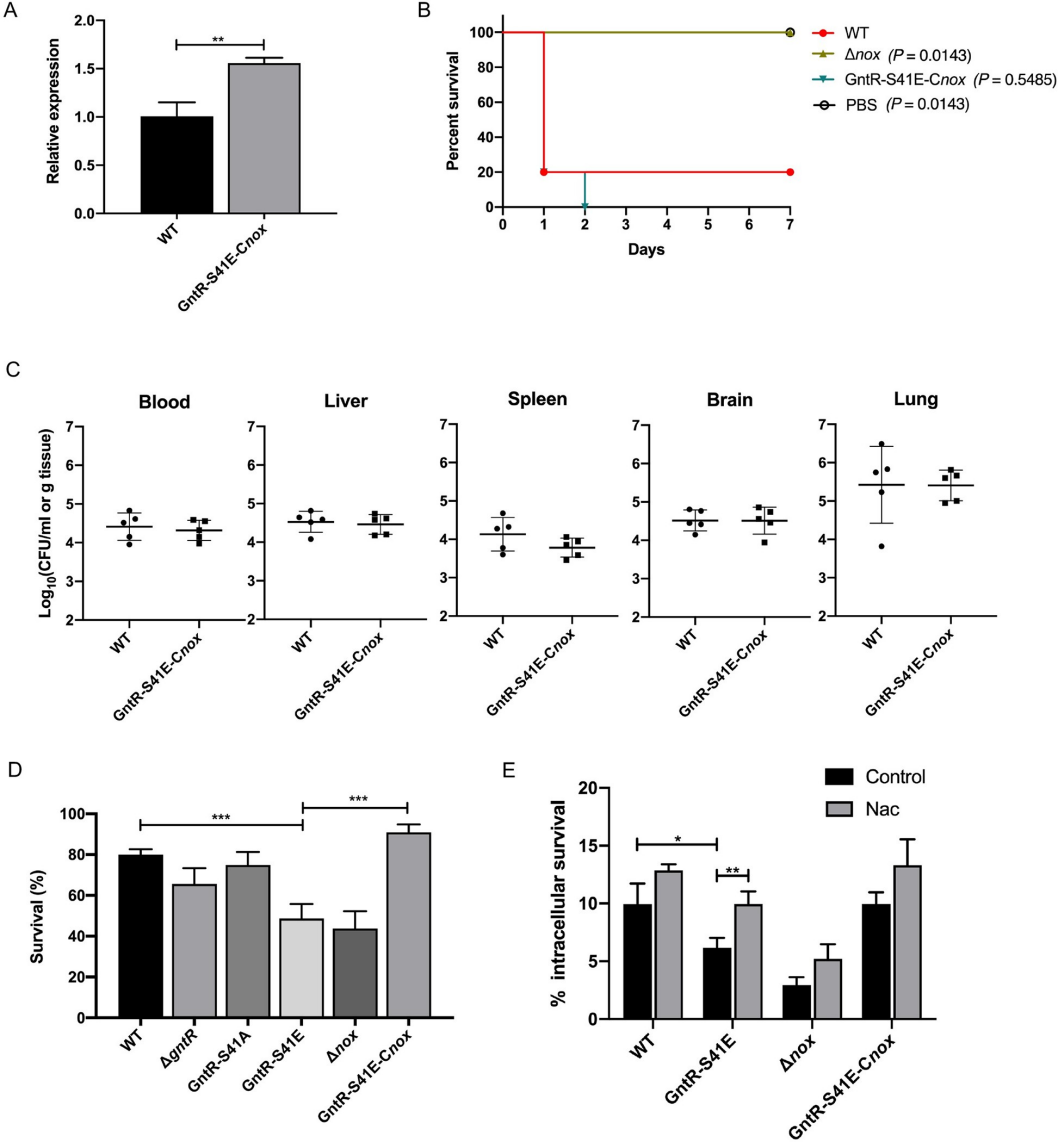
Attenuated virulence and antioxidative capacity of the phosphorylation of GntR are associated with the transcriptional repression of *nox*. (A) The gene of *nox* transcript levels in WT SS2 and GntR-S41E-C*nox* were detected by RT-qPCR. (B) BALB/c mice were challenged by intraperitoneal injection with WT SS2 and GntR-S41E-C*nox* at a dose of 5×10^8^ CFUs/mice (n = 5 mice/group), and the survival time was monitored. (C) Bacterial loads in the blood, lung, liver, spleen, and brain of BALB/c mice were challenged with 5×10^7^ CFUs WT SS2 and GntR-S41E-C*nox* by intraperitoneal injection at 24 hpi (n = 5 mice/group). (D) WT SS2, Δ*gntR*, GntR-S41A, GntR-S41E, Δ*nox*, and GntR-S41E-C*nox* were treated with 30 mM H_2_O_2_ for 30 min, and the number of viable bacteria was subsequently determined by CFU counts. The bacterial survival is expressed as the percentage survival of the initial bacterial count at 0. (E) RAW264.7 cells were infected with 10 MOI WT SS2, GntR-S41E, Δ*no*x, and GntR-S41E-C*nox* for 1 h. After 1 h of antibiotic treatment, the amount of internalized bacteria was regarded as 100% to calculate percent survival. The cells were lysed to release bacteria after 3 h of antibiotic treatment and were serial-diluted in PBS buffer and spread onto THY plates, incubated at 37°C for 16 h. Black bars correspond to the control group, and gray bars correspond to the NAC-treated group. The data shown represent three independent experiments and are presented as the mean ± standard deviations. Statistical analysis was performed using an unpaired *t*-test (A), Long-rank (B), one-way ANOVA (D) and two-way ANOVA (E). *, *P* < 0.05; **, *P* < 0.01; ***, *P* < 0.001.

It has been shown that NOX was involved in bacteria’s anti-oxidant capacity and virulence [23]. Therefore, we speculate that the reduced virulence of the GntR-S41E strain is related to the transcriptional repression of *nox*. We attempted to restore the transcriptional level of *nox* in the GntR-S41E strain. The promoter of *impdh* was selected for the complemented plasmid to exclude the interference of the *nox* promoter. The complemented plasmid was electroporated into GntR-S41E competent cells to obtain the strain GntR-S41E-C*nox*. RT-qPCR detection showed that the transcription level of *nox* was restored in the GntR-S41E-C*nox* strain (Fig 4A). The animal infection experiment showed that the mice lethality rate (Fig 4B) and the colonization efficiency (Fig 4C) of the GntR-S41E-C*nox* strain were restored to the wild-type level. These results suggest that the attenuated virulence of SS2 caused by the STK/GntR phosphorylation pathway is due to the inhibition of *nox* transcription.

### GntR phosphorylation affects anti-oxidant capacity and survival in host macrophages

NOX is involved in bacterial oxidative stress. Therefore, we tested the ability of WT SS2 and *gntR* variants to survive under the condition of oxidative stress. The results showed that the survival rate of the GntR-S41E strain was significantly attenuated after stimulation with 30 mM H_2_O_2_ for 30 min, while the GntR-S41E-C*nox* strain was restored level relative to the wild-type strain (Fig 4D).

Macrophages can produce H_2_O_2_ and other oxidants, which serve to destroy infecting pathogens [24]. We further investigated whether GntR phosphorylation is involved in the intracellular survival of SS2 within RAW264.7 macrophages. Intracellular survival was measured in the absence or presence of N-acetyl-L-cysteine (NAC), a potent ROS inhibitor [25], to investigate the effect of the macrophage oxidative burst on both wild-type and mutants. In the absence of NAC, the intracellular survival of the GntR-S41E strain was markedly decreased compared with the wild-type strain, while GntR-S41E-C*nox* rescued the defect in intracellular survival, and the survival reached the same level as wild-type (Fig 4E). Whereas the number of intracellular GntR-S41E strains recovered from untreated macrophages was significantly lower than that of wild-type, in the presence of NAC, intracellular survival of the GntR-S41E was significantly increased (Fig 4E). NAC treatment partially abrogated its intracellular survival defect, which suggested that SS2 killing by the macrophage oxidative burst is partially counteracted by GntR phosphorylation target gene expression. Our results indicated that the STK/GntR pathway not only decreases the ability of anti-oxidant capacity but also significantly reduces survival in macrophages cell. The phenomenon was related to the inhibition of *nox* transcription.

### The STK-GntR Axis regulates cellular NADH/NAD^+^ ratio

**Fig 5:**
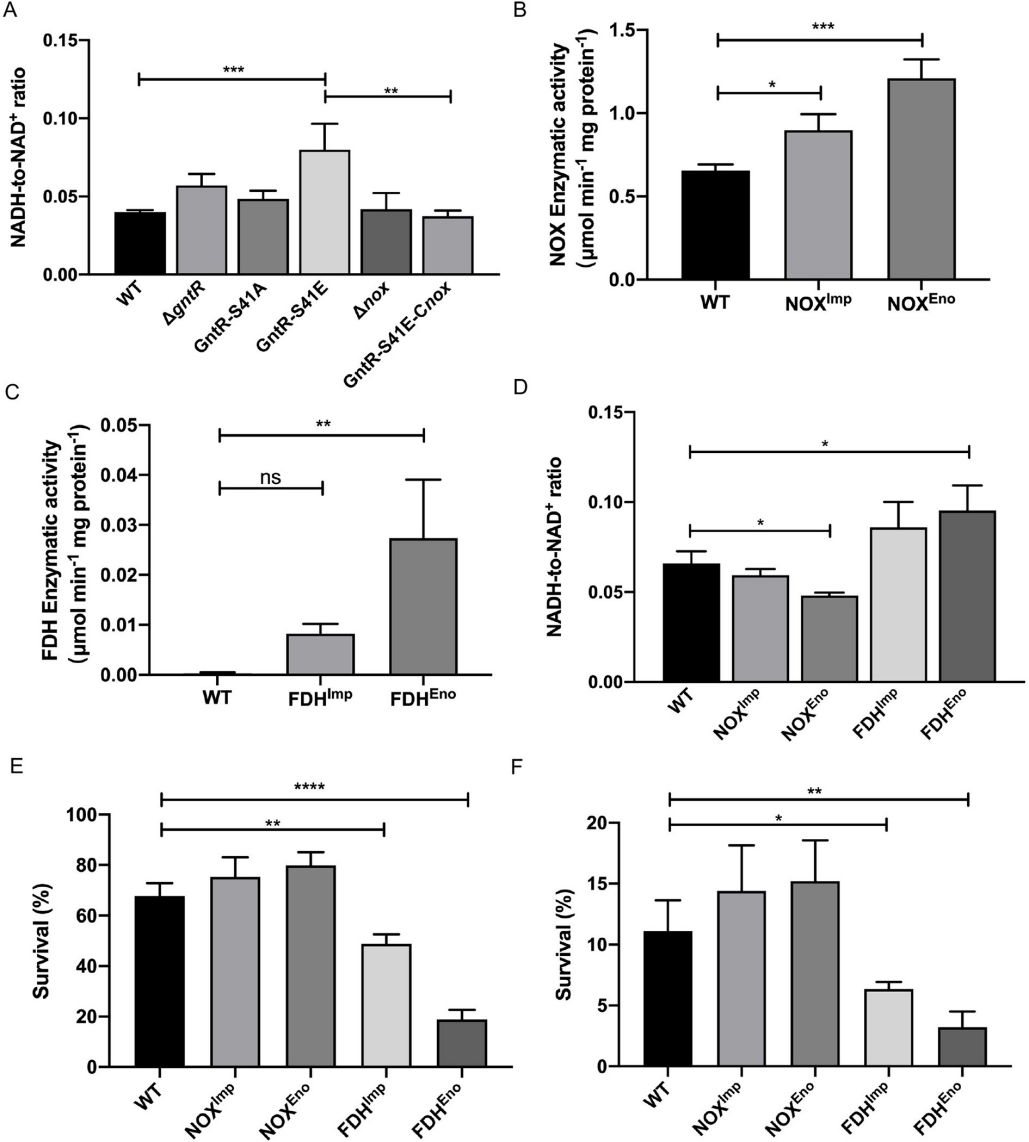
NADH levels affect the anti-oxidant capacity of *S*. *suis*. (A) WT SS2, Δ*gntR*, GntR-S41A, GntR-S41E, Δ*nox*, and GntR-S41E-*nox* were exposed to 1 mM H_2_O_2_ for 1 h, and intracellular levels of NADH/NAD^+^ ratio were measured. (B-C) Preparation of WT SS2, NOX^Imp^, NOX^Eno^, FDH^Imp^, and FDH^Eno^ cells extracts and catalase activity of NOX and FDH. Activities enzymes are expressed as μM per min and mg total protein. (D) WT SS2, NOX^Imp^, NOX^Eno^, FDH^Imp^, and FDH^Eno^ strains were harvested at the log phase, and intracellular NADH/NAD^+^ ratio levels were measured. (E) WT SS2, NOX^Imp^, NOX^Eno^, FDH^Imp^, and FDH^Eno^ strains were treated with 30 mM H_2_O_2_ for 30 min, and detected bacterial survival. (F) RAW264.7 cells were infected with 10 MOI WT SS2, NOX^Imp^, NOX^Eno^, FDH^Imp^, and FDH^Eno^ for 1 h. After 1 h of antibiotic treatment, the amount of internalized bacteria was regarded as 100% to calculate percent survival. The cells were lysed to release bacteria after 3 h of antibiotic treatment, and count the number of viable bacteria and calculate the survival rate. Data shown represent three independent experiments, and are presented as the mean ± standard deviations. Statistical analysis was performed using one-way ANOVA. *, *P* < 0.05; **, *P* < 0.01; ***, *P* < 0.001; ****, *P* < 0.0001; ns, not significant.

The function of the NADH oxidase is to oxidize NADH into NAD^+^, the ratio of which reflects the redox state of a cell [26]. To elucidate if interruption of NOX affects cellular NADH/NAD^+^ levels, WT SS2 and *gntR* variants were challenged with H_2_O_2_, followed by extraction and analysis of NADH and NAD^+^ concentrations. To rule out the effect of bacterial death due to oxidative stimulation, the low-concentration H_2_O_2_ (1 mM) was chosen. GntR-S41E strain resulted in dramatic accumulations of NADH/NAD^+^ ratio by H_2_O_2_, while GntR-S41E-C*nox* strain was restored level relative to the wild-type strain (Fig 5A), indicating that the STK/GntR pathway can lead to the accumulation of NADH/NAD^+^ in bacteria under oxidative stress by inhibiting *nox* transcription. Our results showed that the NADH/NAD^+^ concentrations in Δ*nox* did not increase (Fig 5A), and the survival of Δ*nox* was significantly decreased compared with other strains (S5 Fig). The survival ability of Δ*nox* by 1 mM H_2_O_2_ at different times was detected, and the results showed that the survival of Δ*nox* did not change significantly before 20 min but dramatically decreased at 30 min after the treatment (S6A Fig). The NADH/NAD^+^ ratio gradually increased within 20 minutes and decreased after that (S6B Fig). These observations reveal Δ*nox* could also cause accumulation of NADH/NAD^+^ ratio under oxidative stimulation, but rapidly decreased if cell death.

### Redox cofactor engineering in *S*. *suis*

NADH acts as an electron carrier generated from substrate oxidation and is an important cofactor in diverse redox reactions. The total NADH concentrations determine the reaction activity of the intracellular redox reactions, and the reaction rate is also affected by the ratio of the reduction and oxidation states of these molecules [27]. To explore the effect of the NADH/NAD^+^ ratio on bacteria, in this study, we increased the NADH/NAD^+^ ratio by controlling the expression of the formate dehydrogenase FDH of *Staphylococcus aureus* in *S*. *suis*, adding formate during bacteria culture, and thus catalyzed the oxidation of formate to CO_2_ while increasing NAD^+^ to NADH [28]. We controlled the expression of NADH oxidase in *S*. *suis*, which is highly homologous to *S*. *pneumoniae*, and catalyzed the reduction of O_2_ to H_2_O and negligible formation of H_2_O_2_. Therefore, the NOX-mediated reduction of NADH had no significant effect on ROS generation [29]. The FDH and NOX expression is driven by the promoters of *impdh* [30] and *enolase* [31].

In cell extracts obtained from the collected cells, enhanced activities of both enzymes were detected, indicating that *S*. *suis* cells produce fully functional enzymes (Fig 5B and 5C). In addition, the growth curve of strains was examined, and we determined that carrying the plasmid and adding 10 mM formate to the medium did not affect the growth rate (S7 Fig). We next quantified the NADH/NAD^+^ ratio in strains. We observed the intracellular NADH concentration of the NOX^Eno^ strain with a marked decrease, whereas the FDH^Eno^ strain was significantly increased (Fig 5D).

### Increased NADH levels contribute to the killing of ROS

The electron transport chain becomes hyperactivated, which stimulates the formation of ROS [32,33]. Therefore, we tested the ability of H_2_O_2_ to kill the strains of redox cofactor engineering. The results showed that the survival of the FDH^Eno^ strain was significantly reduced more than thrice, and the ability of resistance to H_2_O_2_ killing of FDH^Imp^ strain was also weakened. The anti-H_2_O_2_ killing ability of NOX^Eno^ was slightly enhanced (Fig 5E). Our results suggest that higher NADH levels decreased resistance to ROS killing. We further evaluated whether NADH/NAD^+^ ratio affects the intracellular survival of SS2 in macrophages, and the result showed that the FDH^Eno^ and FDH^Imp^ strains exhibited significantly reduced intracellular survival in macrophages (Fig 5F).

## Discussion

GntRs are common transcription factors in bacteria, that participate in the regulation of cell motility, bacterial resistance, and virulence [34]. In this study, we found that a GntR is a substrate of STK. The phosphorylation site S41 of GntR was determined using *in vitro* phosphorylation assays (Fig 1B). Subsequently, we constructed deletion (Δ*gntR*), phosphorylation-silencing (GntR-S41A), and phosphorylation-mimicking (GntR-S41E) mutant strains to comprehensively assess the function of GntR. Phosphorylation of GntR by STK resulted in a significant reduction in lethality and pathogenicity in mice (Fig 2A–2G) and antioxidative capacity (Fig 4D), suggesting that STK plays an important role in GntR-mediated gene regulation. The GntR in SS2 was compared to other Streptococci. In *Streptococcus pyogenes* (group A *streptococcus*, GAS) and *Streptococcus agalactiae* (group B *streptococcus*, GBS), the sequence identity of these GntRs is less than 30%, indicating low homology. However, a GntR in *Streptococcus pneumoniae* exhibits considerable sequence homology (56% identity) with the GntR in this study, and the amino acid sequence near the phosphorylation site is highly consistent (S8 Fig). This finding will provide important references for the research of *Streptococcus pneumoniae*.

Domain analysis of GntR using the SMART server revealed that Ser-41 is located in the HTH domain of GntR. Similar regulatory mechanisms of STK-mediated phosphorylation of the HTH domain of protein have been previously reported, such as GraR, CcpA, YvcK, and CovR [13,35–37]. In bacteria, phosphorylation can positively or negatively affect the DNA binding activities of transcription factors to their target genes by adding local negative charges [38]. In *S*. *aureus*, phosphorylation of SarA at Ser-75 in the HTH-DNA-binding domain negatively regulates its DNA-binding activity [39]. Conversely, phosphorylation of SpoVG by STK1 promotes its DNA-binding activity [40]. We performed an EMSA experiment to test the binding of GntR and GntR-S41E to its promoter DNA. The result showed that the GntR-S41E completely lost its ability to bind its promoter DNA (S9 Fig), indicating that GntR phosphorylation negatively affects DNA binding activities.

EMSA results showed that GntR-S41E lost its ability to bind the promoter DNA of *nox* (Fig 3C) and RT-qPCR detection indicated that GntR phosphorylation negatively regulates *nox* transcription, however, we did not find this phenomenon in Δ*gntR*. The implied possible mechanism for this need to be explored further. We hypothesized that the reduced anti-oxidant capacity and virulence of GntR phosphorylation strains were associated with the transcriptional repression of *nox*. The complementation strain GntR-S41E-C*nox* restored its antioxidative capacity and virulence to the wild-type strain (Fig 4B and 4C). These results suggest that the transcriptional repression of *nox* is the key reason for the weakened anti-oxidant capacity and virulence of the GntR-S41E strain.

In *Mycobacterium tuberculosis*, interruption of NADH hydrolytic activities resulted in dramatic accumulations of NADH by H_2_O_2_ and increased oxidative stress susceptibility [41]. In this study, the GntR-S41E strain also causes the accumulation of NADH during oxidative stress because of the transcriptional repression of NADH oxidase (Fig 5A). Higher NADH levels lead to increased intracellular ROS and enhanced lethality of antibiotics in *Pseudomonas aeruginosa* [27]. To verify whether higher NADH levels are the main cause of the increased oxidative stress susceptibility, four NADH/NAD^+^ ratio control strains were constructed. Our data suggested that higher NADH levels reduced anti-oxidant capacity and intracellular survival of SS2 (Fig 5E and 5F).

**Fig 6:**
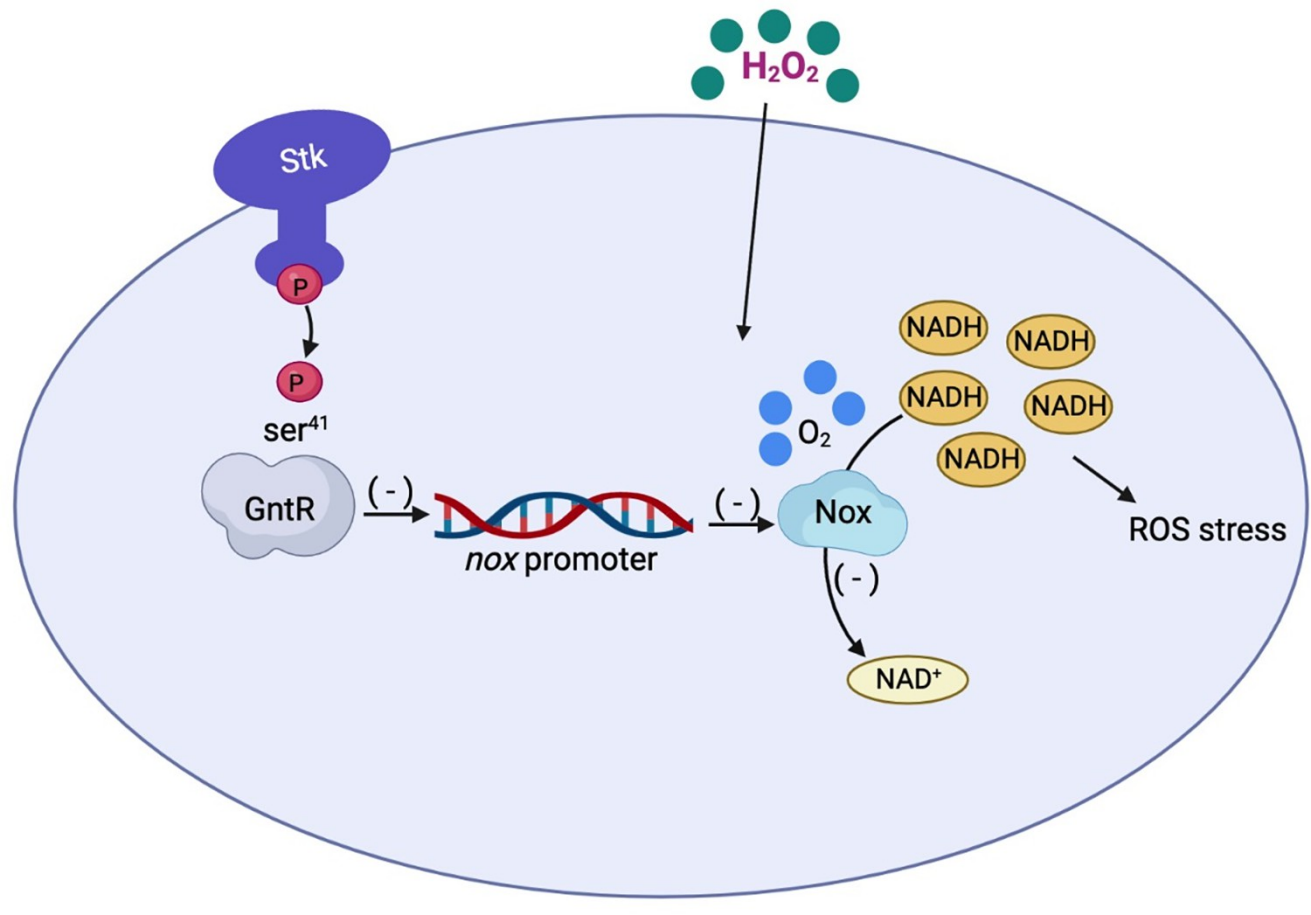
Proposed model for STK/GntR pathway that the impacts on oxidative stress and intracellular survival mechanisms in *S*. *suis* exposed to an oxidizing environment.

Relatively little is known about the regulation of GntR and by demonstrating its phosphorylation by STK we here describe a new regulation mechanism for this phosphokinase; that is, SS2 STK repressed the transcription of *nox* through the phosphorylation of GntR, which led to the accumulation of NADH under oxidative stress and increased amplified ROS killing (Fig 6). The impaired anti-oxidative stress may be the key reason for the reduced virulence of SS2. This finding provides a basis for elucidating the regulatory mechanism of STK in SS2.

## Materials and methods

### Ethics statement

All animal experiments were approved by the Laboratory Animal Welfare and Ethics Committee of Nanjing Agricultural University, China (approval number NJAU.No20210510065 and NJAU.No20211005144). The Chinese National Laboratory Animal Guideline for Ethical Review of Animal Welfare adhered to animal care and protocol.

### Bacterial strains, plasmids, cell lines, and grown conditions

Bacteria strains and plasmids used in this study are listed in the S3 Table. The SS2 strain ZY05719 was isolated from pigs that died during the outbreak of the streptococcal disease in Sichuan Province, China. SS2 was grown in THY (Todd-Hewitt Broth [THB, Becton, Franklin Lakes, NJ, USA] containing 2% yeast extract [Oxoid Ltd., UK]) or in THA (THY containing 1.5% agar). *E*. *coli* was cultured in Lysogeny broth (LB, Oxoid Ltd.) or LB supplemented with 1.5% agar. When necessary, antibiotics were added to the media at the following concentrations: spectinomycin (Spc, *E*. *coli* 50 μg/mL, SS2 100 μg/mL), ampicillin (Amp, 100 μg/mL), kanamycin (Kan, 50 μg/mL). RAW264.7 was cultured in DMEM containing 10% fetal bovine serum at 37°C and 5% CO_2_.

### Strain construction

Deletions and insertion were generated using homologous recombination in bacteria as described previously [42]. The fragments upstream and downstream of *gntR* were amplified from the ZY05719 genome using the primers Δ*gntR*-F1/Δ*gntR*-R1 and Δ*gntR*-F2/Δ*gntR*-R2. PCR products were mixed 1:1, and primer pair Δ*gntR*-F1/Δ*gntR*-R2 were subjected to fusion PCR amplification. The fusion fragment was cloned into temperature-sensitive *S*. *suis* and *E*. *coli* shuttle vector pSET4s using ClonExpress II One Step Cloning Kit (Vazyme Biotech Co., China) to obtain the vector pSET4s-*gntR*. The plasmids of pSET4s-*gntR*-S41A, pSET4s-*gntR*-S42A, pSET4s-*gntR*-T44A, pSET4s-*gntR-*S41E, and pSET4s-*nox* were obtained similarly. The pSET4s-*gntR* and pSET4s-*nox* were electroporated into ZY05719 using the Gene Pulser XCell electroporation system (Bio-Rad, Hercules, CA, USA) (voltage: 2300 V, capacitance: 25 μF, resistance: 200 Ω, cuvette: 1 mm), and Δ*gntR* and Δ*nox* were carried out as described in the literature [42]. The pSET4s-gntR-S41A, pSET4s-*gntR*-S42A, pSET4s-*gntR*-T44A, and pSET4s-gntR-S41E were electroporated into Δ*gntR*, and the in-situ mutant strain of *gntR* was obtained by the above method.

The complementary strain of the Δ*gntR* mutant was constructed as follows. The *gntR* fusion *flag* fragment was amplified from the ZY05719 genome using the primers CΔ*gntR*-*flag*-F/CΔ*gntR*-*flag*-R, then cloned into pSET2. The recombinant plasmid was electroporated into Δ*gntR*. Construction of NOX and FDH overexpression strains was done as follows. Using SS2 ZY05719 and Staphylococcus aureus MSHR1132 genome as templates, respectively, amplified the *nox* and *fdh* sequence and fused the two sequences into the promoter sequences of *impdh* and *enolase*, respectively. Then cloned into the pSET2 plasmid and electroporated into ZY05719. All primers are listed in the S4 Table.

### Protein expression and purification and polyclonal antibody preparation

The gene sequence of *stk* was amplified from the ZY05719 genome using the primers nSTK-F/nSTK-R and cloned into pGEX4T-1. The gene sequence of *gntR* and *nox* were amplified using the primers GntR-F/GntR-R and NOX-F/NOX-R and cloned into pET28a. Using the primers GntR-F/GntR-S41A-R1(P) and GntR-S41A-F2/GntR-R, respectively, the fragments of *gntR* were amplified. Two fragments were fused using the primer pair GntR-F/GntR-R and cloned into pET28a, and an expression plasmid with Ser-41 to Ala was obtained. The same method was used to obtain pET28a-GntR-S42A, pET28a-GntR-T44A, and pET28a-GntR-S41E. All the above plasmids were transformed into *E*. *coli* BL21 (DE3). GST-tagged proteins were purified using GST-tag purification resin (Beyotime, Shanghai, China), and His-tagged proteins were purified using HisTrap HP (5 mL; GE Healthcare, Piscataway, NJ, USA). BALB/c mice were immunized by subcutaneous injection of 100 μg of the purified GntR protein and NOX protein emulsified with adjuvants. Serum was collected from mice 10 days after the third immunization.

### *In vitro* phosphorylation assays

*In vitro* phosphorylation was carried out with 5 μg of purified recombinant substrate protein and 15 μg of purified nSTK in phosphorylation buffer (100 mM Tris-HCl pH 8.0, 10 mM MgCl_2_, 25 mM NaCl, 100 mM ATP, 1 mM DTT) for 2 h at 37°C. To detect nSTK autophosphorylation, only nSTK protein was added. Samples were separated by standard Tris-glycine-SDS polyacrylamide gel electrophoresis (PAGE) gels and Phos-tag-SDS polyacrylamide gels containing 100 μM phos-tag solution (ApexBio Technology LLC, Houston, TX, USA) and 200 μM MnCl_2_. Separated proteins were visualized with Coomassie Brilliant Blue staining.

### *In vivo* phosphorylation assays

WT SS2 and Δ*stk* were grown in THY to mid-log phase, pelleted, and resuspended in 0.01 M PBS containing lysozyme (1mg/mL), protease inhibitor cocktail (1:100), and phosphatase inhibitor cocktail (1:100). After 1 h incubation at 37°C, sonication was performed for 5 cycles (5 min: 30 s on, 30 s off). Following sonication, cell debris was removed by centrifugation at 12,000 g for 30 min at 4°C. The supernatant was immunoprecipitated using GntR polyclonal antibody crosslinked to ProtreinA/G agarose (Beyotime, Shanghai, China) at 4°C overnight. After the agarose protein complexes were washed thrice in PBS buffer, the agarose was boiled for 10 min in SDS-PAGE protein loading buffer. Western blot analysis was performed using GntR polyclonal antibody and anti-phospho-(Ser/Thr) antibody (Abcam, Cambridge, MA, USA).

### Animal experiments

Four weeks old female BALB/c mice were purchased from the Comparative Medicine Center of Yangzhou University (Yangzhou, China). To investigate the survival curves of mice, the mice were randomized to experimental groups and were inoculated with WT SS2 or *gntR* variants at a dose of 5×10^8^ CFUs via intraperitoneal injection. An equal volume of PBS was used as a control. Survival rates and clinical conditions were monitored for 7 days after the challenge. To compare the colonization ability of WT SS2 or *gntR* variants in BALB/c mice, bacteria were injected at a dose of 5×10^7^ CFUs. The bacterial loads in the liver, spleen, lung, brain, and blood were determined 24 h post-infection after the bacterial injection. For histopathological analysis, the lung and spleen of infected BALB/c mice were fixed and embedded in paraffin, and the blocks were sectioned and stained with hematoxylin-eosin. Observe the lesions under the microscope. Microscopic lesions were evaluated in from five random fields of each tissue section in a blinded manner and scored as follows: 0, no lesions; 1, minimal; 2, mild; 3, moderate; 4, severe.

### Susceptibility to oxidative stress

The ability of bacteria to survive in oxidative stress was determined by the method of Roy S [43] with minor modifications. Briefly, SS2 cultured to OD_600_ = ~0.6. After centrifugation, cells were washed a third time in PBS and resuspended in 1 mL PBS containing 30 mM H_2_O_2_ for 30 min at 37°C. The survival rate was calculated based on the number of colonies at the start of the treatment. The experiment was performed three times.

### EMSA

The binding of GntR protein to the *nox* promoter was detected using native polyacrylamide gel electrophoresis. The online tool BProm program (SoftBerry) was used to predict the promoter sequence of *nox*, and the product was purified after PCR amplification. DNA probes were incubated with GntR or point mutant protein in binding buffer (10 mM Tris-base, 50 mM KCl, 5 mM MgCl_2_, 1 mM DTT, 0.05% Nonidet P-40, 2.5% glycerol, pH 7.5) for 30 min at 37°C. Protein-DNA complexes were loaded onto a 6% native polyacrylamide gel, electrophoresed in 0.5 × TBE buffer at 200 V for 30 min, and visualized by ethidium bromide staining.

### ChIP assay

CΔ*gntR-flag* was fixed with a final concentration of 1% formaldehyde for 10 min, and crosslinking was terminated with a final concentration of 0.125 M glycine. The cell was resuspended in lysis buffer and was sonicated to fragment chromosomal DNA in the range of 0.5–1.0 kb. After centrifugation, the anti-Flag antibody (Engibody, WI, USA) or negative mouse IgG (Beyotime, Shanghai, China) was added to the input fraction for immunoprecipitation. They were incubated overnight at 4°C with rotation, and proteinA/G beads (Beyotime, Shanghai, China) were added to the mixture and incubated for 2 h. The immunoprecipitated complexes were collected and washed twice with ChIP wash buffer and twice with TE solution, then eluted with ChIP elution solution. The collected eluate was added with proteinase K and RNase (TAKARA, Dalian, China) to remove protein and RNA, and the DNA was recovered using a DNA fragment pure kit (OMEGA, USA). The recovered DNA was used as output for PCR analysis.

### RNA isolation and RT-qPCR assays

Briefly, total RNA was extracted from bacteria using Total RNA Extraction Reagent (Vazyme Biotech Co., China) according to the manufacturer’s protocol. RNA samples were then treated with HiScriptII Q RT SuperMix for qPCR (+gDNA wiper) (Vazyme Biotech Co., China) to remove any containing genomic DNA and synthesize cDNA. cDNA was used as a temple for SYBR Green-based qPCR using ChamQ Universal SYBR qPCR Master Mix (Vazyme Biotech Co., China) and a 7300 Real-Time PCR System (Applied Biosystems, Foster City, California, USA). The primer sequences are shown in the S4 Table. The *gapdh* gene was used as an internal control, and the 2^-ΔΔCt^ method was used to calculate the fold changes in transcript levels. Assays were performed in triplicate.

### β-Galactosidase activity assays

The promoter of *nox* was amplified using the primer pairs listed in the S4 Table. The resulting product was fused to the promoterless lacZ report vector pTCV-lacZ, and then electroporated into WT SS2, Δ*gntR*, GntR-S41A, and GntR-S41E. Measurement of β-galactosidase activities refers to Miller’s method [44].

### Intracellular survival assays

The intracellular survival assays of *S*. *suis* were performed as reported previously with modification [45]. Bacteria were co-cultured with RAW264.7 cell monolayers at an initial multiplicity of infection (MOI) of 10 for 1 h at 37°C and 5% CO_2_. For the NAC group, RAW264.7 cells were treated with 5 mM NAC at the same time that bacterial infection. After 1 h, cells were washed thrice with PBS, and 1 mL of DMEM containing 100 μg/mL gentamicin and 10 μg/mL penicillin were added to the cells for 1 h and 3 h. After a time, cells were washed three times with PBS and were treated with sterile water to lyse. The number of CFUs of intracellular bacteria was determined by plating a serial dilution of the lysates on THY agar plates. The intracellular survival rate was calculated as CFU_3h_/CFU_1h_ × 100%. All experiments were repeated thrice, each time with triplicate wells.

### Preparation of cell extracts and *in vitro* enzymatic assays

SS2 was cultured in THY medium (add 10 mM sodium formate if necessary) to OD_600_ = ~0.6, harvested by centrifugation (5 min; 6,000 rpm; 4°C), and resuspended in ice-cold 50 mM potassium phosphate buffer (pH 7.4) containing 1 × protease inhibitors. Cells were sonicated and then centrifuged (10 min; 12,000 rpm; 4°C) to collect protein extracts. The total protein concentration of the extracts was determined by the Bradford method.

The enzymatic activities were determined by monitoring spectrophotometrically the oxidation of NADH (for NOX) and the reduction of NAD^+^ (for FDH) at λ = 340 nm (A_340_ nm). For NADH oxidase, 90 μL of NOX buffer (50 mM potassium phosphate buffer [pH 7.4], 0.2 mM NADH, and 0.3 mM EDTA) were mixed with 10 μL of the cell extract, and A_340_ nm was automatically recorded every 30 s in a plate reader (Multimode microplate reader, TECAN, Switzerland) at 30°C. For formate dehydrogenase, 90 μL of FDH buffer (50 mM potassium phosphate buffer [pH 7.4], 150 mM sodium formate, and 2 mM NAD^+^) were mixed with 10 μL of the cell extract and incubated in the plate reader as indicated above. In both cases, the maximum linear rate was obtained by plotting A_340 nm_ min^-1^, and the specific activity for each enzyme was calculated by an extinction coefficient of 6.22 mM^-1^ cm^-1^ for NADH. One unit of enzyme activity (IU) was defined as the amount of enzyme catalyzing the formation/oxidation of 1 μM of NADH per mg protein/min.

### Quantification of intracellular NAD(H)

Intracellular levels of NADH/NAD^+^ were measured using the NAD(H) Quantification Kit (Solarbio Science & Technology Co. Beijing). For NAD^+^ extraction, 1 billion bacteria were collected, 0.5 mL acidic extract was added, and boiled for 5 minutes after ultrasonication. After cooling in an ice bath, samples were centrifuged at 10,000 g at 4°C for 10 min. 200 μL of the supernatant was transferred to a new tube and mixed with an equal volume of alkaline extract. Samples were centrifuged at 10,000 g at 4°C for 10 min, and the supernatant was kept on ice until needed. The order of using acidic and alkaline extract was reversed for NADH extraction, and the other steps were the same as for NAD^+^ extraction. The concentrations of NADH and NAD^+^ in the samples were determined according to the manufacturer instructions and normalized to cell number.

### Data analysis

GraphPad Prism 8.0 software (La Jolla, CA, USA) was used for data analysis. Bars represent the mean ± standard deviation (SD) calculated from at least three biological replicates. An unpaired *t*-test (comparison of 2 groups), Long-rank (comparison of survival curves), and one-way ANOVA or two-way ANOVA were used to analyze the statistical significance. *P* < 0.05 were considered statistically significant.

## Supporting information

S1 Fig The mass spectrum of the phosphorylated peptide to LFNVSSphosITVIR shows that GntR is phosphorylated at Ser-42. (PDF)

S2 Fig Construction of Δ*gntR*, GntR-S41A, and GntR-S41E. (A) Δ*gntR* was identified by PCR using primers Δ*gntR*-F1/Δ*gntR*-R2 (OUT) and GntR-F/GntR-R (IN). (B) The GntR-S41A and GntR-S41E strains were determined by PCR-based Sanger sequencing. (C) The THY medium’s growth curves of WT SS2, Δ*gntR*, GntR-S41A, and GntR-S41E were measured with a spectrophotometer at 600 nm.
(PDF)

S3 Fig RT-qPCR analyses relative transcript levels of *gntR* in WT SS2 at different growth stages. (PDF)

S4 Fig Construction of Flag-tagged GntR strain. (A) WT SS2 and GntR-S41E-C*nox* were detected by Western blot with an anti-FLAG antibody. (B) The fusion of the Flag to GntR does not affect its function. One-way ANOVA was used to test the significance of the data. *, *P* < 0.05; ns, not significant.
(PDF)

S5 Fig Survival rates of WT SS2, Δ*gntR*, GntR-S41A, GntR-S41E, Δ*nox*, and GntR-S41E-*nox* after treatment at 1 mM H_2_O_2_ for 1 h. one-way ANOVA was used to test the significance of the data. ****, P < 0.0001.
(PDF)

S6 Fig Detection of the effect of Δ*nox* on oxidative stres*s*. Δ*nox* was exposed to 1 mM H_2_O_2_ for 1 h, and the survival rate (A) and intracellular levels of NADH/NAD^+^ ratio (B) were measured.
(PDF)

S7 Fig Growth of WT SS2, NOX^Imp^, NOX^Eno^, FDH^Imp^, and FDH^Eno^ in THY medium was measured with a spectrophotometer at 600 nm. (PDF)

S8 Fig Sequence alignment of GntR proteins in *Streptococcus suis* and *Streptococcus pneumoniae*. (PDF)

S9 Fig EMSA experiments using GntR and GntR-S41E proteins and *gntR* promoter DNA. (PDF)

S1 Table Phosphoproteomics results of GntR in WT SS2 and Δ*stk*. (XLSX)

S2 Table Expression levels of genes in Δ*gntR* compared to WT SS2. (DOCX)

S3 Table Strains and plasmids used in this study. (DOCX)

S4 Table Primer used in this study. (DOCX)

S1 Data Excel spreadsheet containing the numerical data and statistical analysis for Figure panels 2A, 2B-2F, 2H-2I, 3E, 3G, 4A, 4B, 4C, 4D, 4E, 5A, 5B-5C, 5D, 5E, 5F, S3, S4B, S5, and S6A-S6B. (XLSX)
