# Supplementary figures and images for: Phosphorylation of GntR reduces Streptococcus suis oxidative stress resistance and virulence by inhibiting NADH oxidase transcription
Source: PLoS Pathog. 2023 Mar 13;19(3):e1011227. doi: 10.1371/journal.ppat.1011227 (PMC10010549; doi:10.1371/journal.ppat.1011227)

| Raw file       | Scan number | Mass analyzer | Score  | m/z      | Proteins   |
|----------------|-------------|---------------|--------|----------|------------|
| SA156LPST_WT_1 | 40733       | FTMS          | 122.64 | 664.8523 | A0A0H3MTN4 |

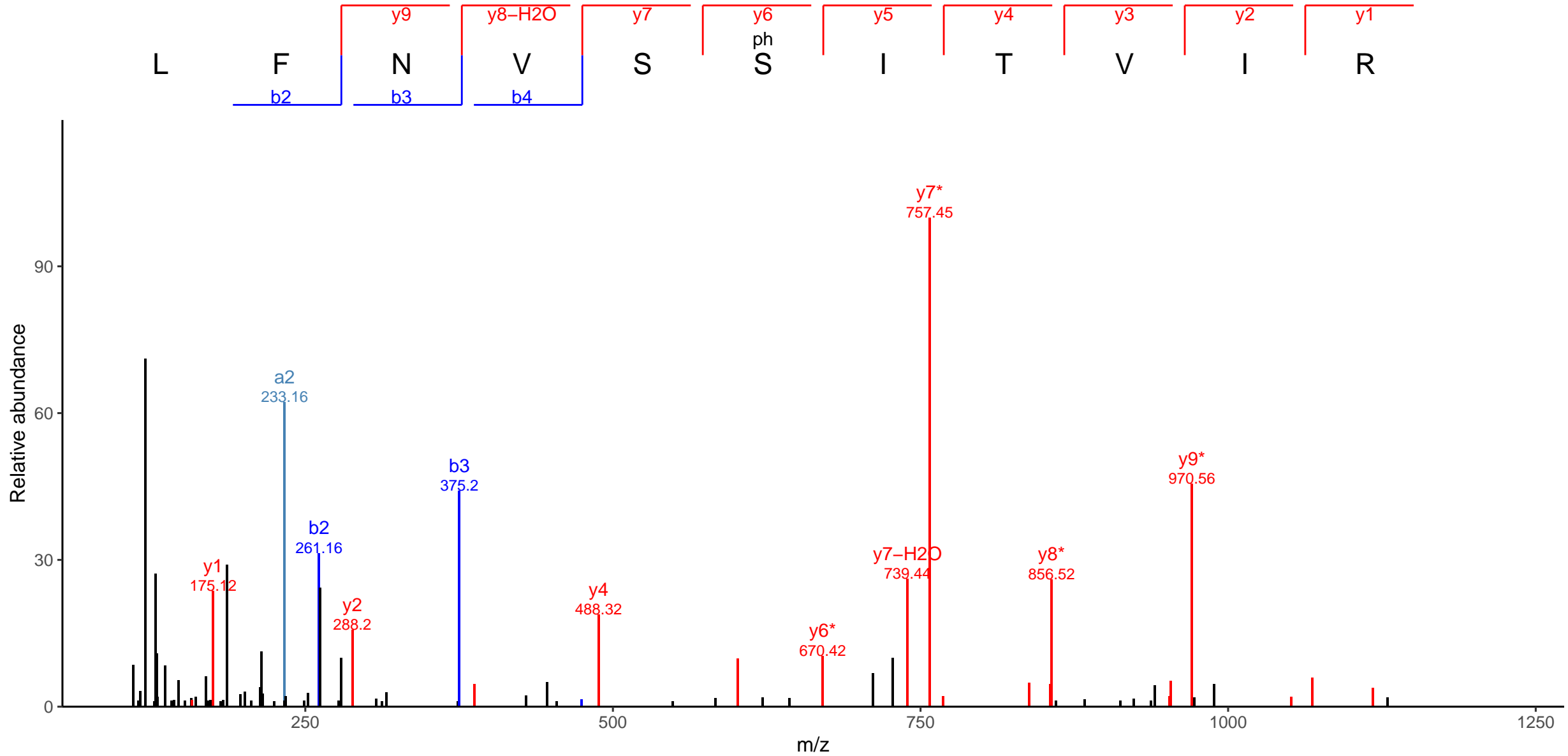

Supplement: S1 Fig — (PDF) [file ppat.1011227.s001.pdf]

A

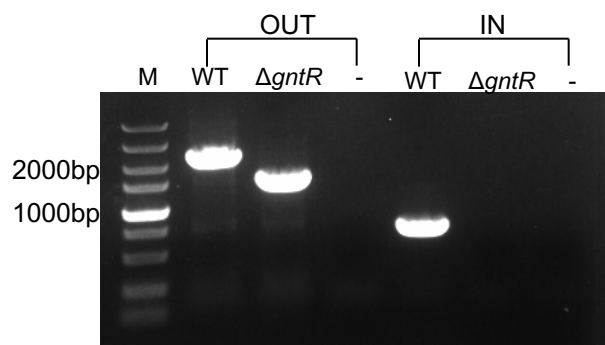

B

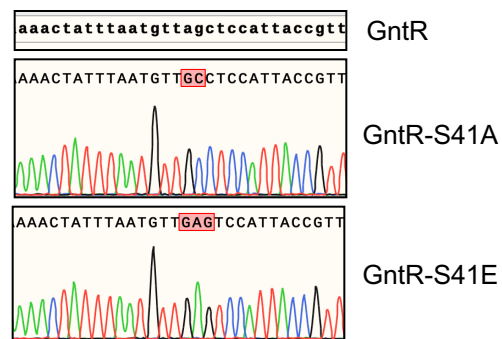

C

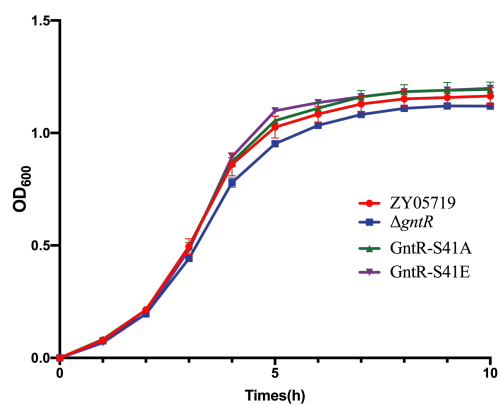

Supplement: S2 Fig — (A) ΔgntR was identified by PCR using primers ΔgntR-F1/ΔgntR-R2 (OUT) and GntR-F/GntR-R (IN). (B) The GntR-S41A and GntR-S41E strains were determined by PCR-based Sanger sequencing. (C) The THY medium’s growth curves of WT SS2, ΔgntR, GntR-S41A, and GntR-S41E were measured with a spectrophotometer at 600 nm. (PDF) [file ppat.1011227.s002.pdf]

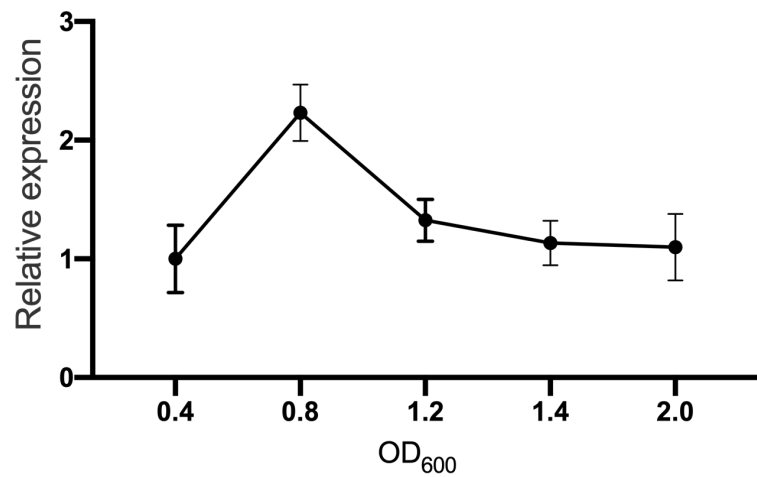

Supplement: S3 Fig — (PDF) [file ppat.1011227.s003.pdf]

A

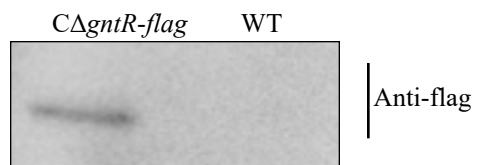

B

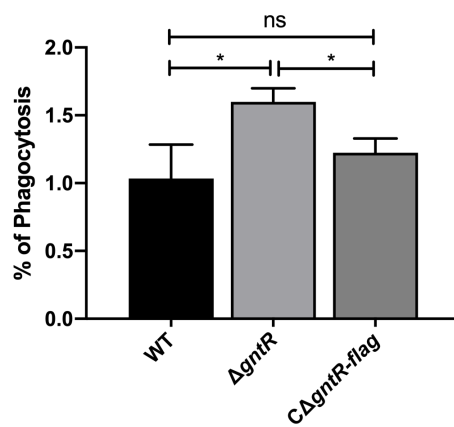

Supplement: S4 Fig — (A) WT SS2 and GntR-S41E-Cnox were detected by Western blot with an anti-FLAG antibody. (B) The fusion of the Flag to GntR does not affect its function. One-way ANOVA was used to test the significance of the data. *, P < 0.05; ns, not significant. (PDF) [file ppat.1011227.s004.pdf]

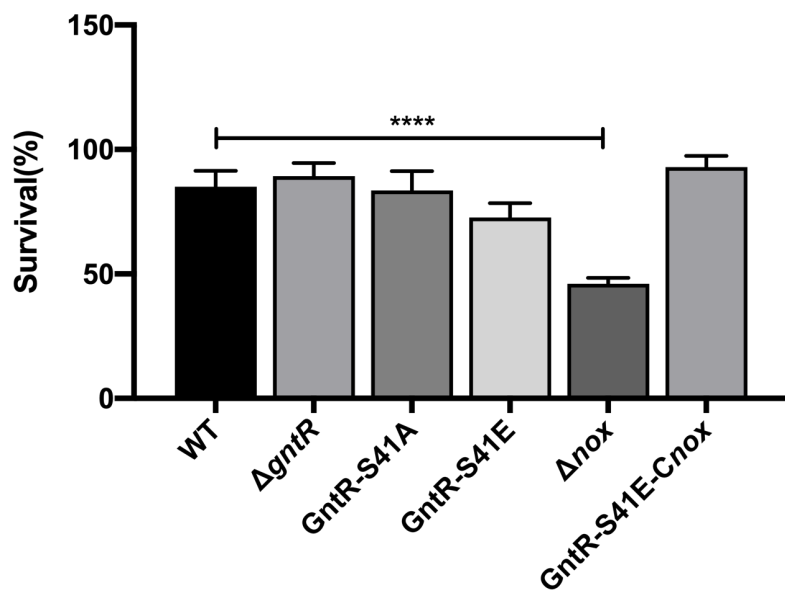

Supplement: S5 Fig — one-way ANOVA was used to test the significance of the data. ****, P < 0.0001. (PDF) [file ppat.1011227.s005.pdf]

A

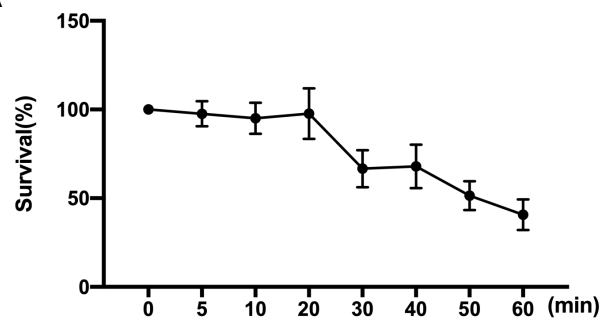

B

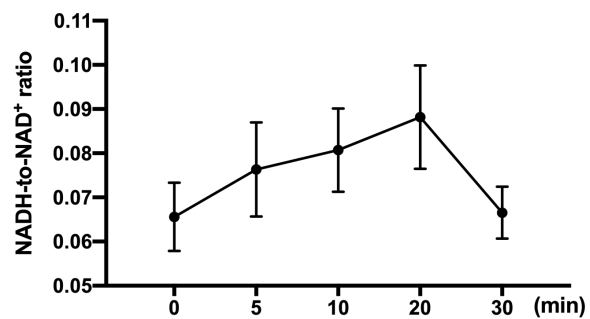

Supplement: S6 Fig — Δnox was exposed to 1 mM H2O2 for 1 h, and the survival rate (A) and intracellular levels of NADH/NAD+ ratio (B) were measured. (PDF) [file ppat.1011227.s006.pdf]

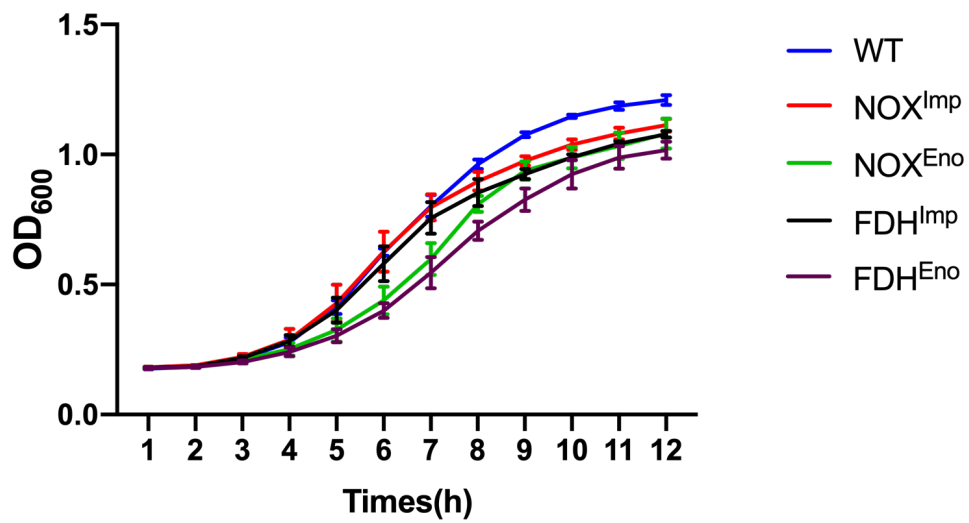

Supplement: S7 Fig — (PDF) [file ppat.1011227.s007.pdf]

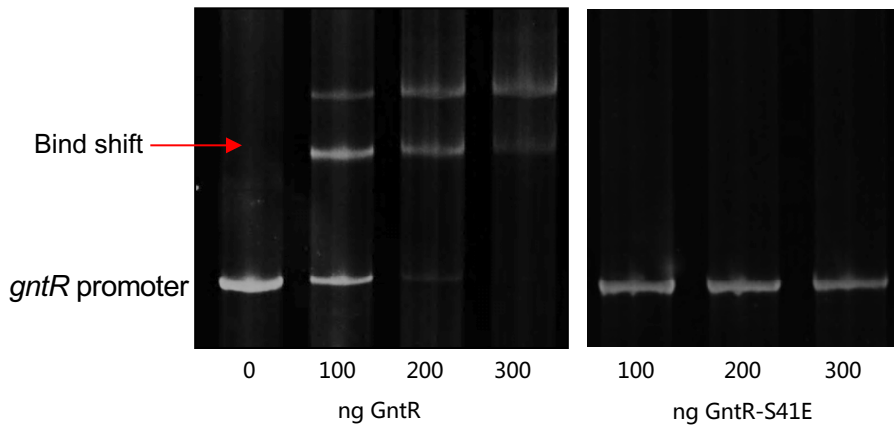

Supplement: S9 Fig — (PDF) [file ppat.1011227.s009.pdf]
