# Supplementary material for: Phosphorylation of GntR reduces Streptococcus suis oxidative stress resistance and virulence by inhibiting NADH oxidase transcription
Source: PLoS Pathog. 2023 Mar 13;19(3):e1011227. doi: 10.1371/journal.ppat.1011227 (PMC10010549; doi:10.1371/journal.ppat.1011227)
Supplement: S8 Fig — (PDF) [file ppat.1011227.s008.pdf]

```
                1        10        20        30        40        50
S.suis        .........MKVPKYQLIQNDLRQQIVSGKFENGDKFYTESELTKLFNVSSITVIRAVNE
S.pneumoniae  MEKENRGKNMAIPKYQYIKDELKNKIISGQFSSGDKFYTEAELISMYDVSSITVVRALND

                      60        70        80        90       100       110
S.suis        LVKDGYLVRQQGKGTFVSRSRKGRLVEFSDIEIFPMDKDKVTVLSCEKGNKPDILEKLNL
S.pneumoniae  LAKDGYIVRQQGKGTFVSRARKHKLVEFSDIELFNAKDDKVTVLSIERGNKLVYLEKLGL

                     120       130       140       150       160       170
S.suis        DKNEFYYKIVRVRAAEDTPYIFHNSYIPQRYIQNPDAPLEHYQSIYQRFKLDYNIHMSEE
S.pneumoniae  RGDQFYYKIERIRESNGVVYIYHTSYIPEQYINANYPNLEYYSSIYNRFKLDYHIHMNDE

                     180       190       200       210       220       230
S.suis        PFVETNEIVSPCPKEVATHLKLKATEPAVLQNKTTTNSTSGEVMEYTETYKHWKYYKFEI
S.pneumoniae  HFEEINEIVFPTPEHAASVLGVDEQFPTVLQTKITKLESTGQVLEYSETYKRSDYYKIKF

S.suis        TANHR..
S.pneumoniae  ISCDRDH
```
