# Supplementary material for: Phosphorylation of GntR reduces Streptococcus suis oxidative stress resistance and virulence by inhibiting NADH oxidase transcription
Source: PLoS Pathog. 2023 Mar 13;19(3):e1011227. doi: 10.1371/journal.ppat.1011227 (PMC10010549; doi:10.1371/journal.ppat.1011227)
Supplement: S2 Table — (DOCX) [file ppat.1011227.s011.docx]

**Table S2.** Expression levels of genes in Δ*gntR* compared to WT SS2

| Code for ORF | log2Fold Change  (ΔgntR/WT) | p-value | Functional annotation |
| --- | --- | --- | --- |
| ZY05719_03505 | 3.321928095 | 0.003324947 | aquaporin family protein |
| ZY05719_02225 | 3.169925001 | 0.006321756 | PTS system mannose/fructose/N-acetylgalactosamine-transporter subunit IIB |
| ZY05719_01105 | 2.976792609 | 6.06E-191 | tagatose-6-phosphate kinase |
| ZY05719_03565 | 2.584962501 | 0.004306683 | Crp/Fnr family transcriptional regulator |
| ZY05719_07430 | 2.107294013 | 4.39E-36 | hypothetical protein |
| ZY05719_06600 | 1.965234582 | 2.64E-19 | hypothetical protein |
| ZY05719_09730 | 1.933181893 | 2.44E-48 | tRNA adenosine deaminase TadA |
| ZY05719_08375 | 1.688055994 | 0.000632826 | hypothetical protein |
| ZY05719_01110 | 1.533530624 | 2.62E-120 | DNA repair protein |
| ZY05719_02210 | 1.525163942 | 0 | GntR family transcriptional regulator |
| ZY05719_04250 | 1.356594332 | 1.81E-298 | phosphomannomutase/phosphoglucomut-ase |
| ZY05719_06595 | 1.346705668 | 5.56E-43 | ABC transporter ATP-binding protein |
| ZY05719_03930 | 1.286136976 | 1.62E-89 | GMP reductase |
| ZY05719_06440 | 1.271340617 | 2.90E-77 | TIGR02206 family membrane protein |
| ZY05719_04060 | 1.167894466 | 5.19E-20 | excalibur calcium-binding domain-containing protein |
| ZY05719_06590 | 1.134243313 | 1.56E-271 | FtsX-like permease family protein |
| ZY05719_01060 | 1.094650376 | 2.60E-86 | SIS domain-containing protein |
| ZY05719_01130 | 1.041222663 | 1.13E-07 | right-handed parallel beta-helix repeat-containing protein |
| ZY05719_03745 | 1.018378529 | 0.0001009 | hypothetical protein |
| ZY05719_07605 | -1.019365325 | 2.03E-09 | ABC transporter ATP-binding protein |
| ZY05719_09670 | -1.02653307 | 9.76E-33 | MarR family transcriptional regulator |
| ZY05719_07115 | -1.034765418 | 0.000206221 | type II toxin-antitoxin system RelE/ParE family toxin |
| ZY05719_07070 | -1.044482436 | 6.82E-39 | PTS glucose transporter subunit IIA |
| ZY05719_03535 | -1.097628126 | 0 | FAD-dependent oxidoreductase |
| ZY05719_02345 | -1.097631319 | 1.90E-57 | class C sortase |
| ZY05719_06820 | -1.103062006 | 3.74E-137 | large conductance mechanosensitive channel protein MscL |
| ZY05719_08715 | -1.123206279 | 0 | dihydrolipoamide acetyltransferase |
| ZY05719_00690 | -1.137503524 | 0.003580913 | hypothetical protein |
| ZY05719_01925 | -1.137503524 | 4.11E-06 | endonuclease/exonuclease/phosphatase family protein |
| ZY05719_04235 | -1.141355849 | 9.43E-21 | AAA family ATPase |
| ZY05719_07065 | -1.213955584 | 9.04E-50 | glycoside hydrolase family 1 protein |
| ZY05719_05765 | -1.224215157 | 1.89E-39 | aconitate hydratase AcnA |
| ZY05719_04575 | -1.229133999 | 2.31E-15 | ATP-binding protein |
| ZY05719_02400 | -1.233603318 | 5.85E-35 | GNAT family N-acetyltransferase |
| ZY05719_00185 | -1.259920626 | 6.95E-108 | rod shape-determining protein MreC |
| ZY05719_05275 | -1.282035368 | 5.25E-07 | hypothetical protein |
| ZY05719_00650 | -1.30256277 | 2.27E-09 | tRNA-Leu |
| ZY05719_08710 | -1.312517328 | 0 | dihydrolipoyl dehydrogenase |
| ZY05719_09480 | -1.358453971 | 3.73E-07 | radical SAM protein |
| ZY05719_04585 | -1.36923381 | 0.004404975 | ABC transporter permease |
| ZY05719_06680 | -1.36923381 | 0.004404975 | PTS transporter subunit EIIC |
| ZY05719_06100 | -1.374124306 | 6.07E-13 | DUF2304 domain-containing protein |
| ZY05719_01650 | -1.398549376 | 0.005094683 | zinc ABC transporter substrate-binding protein |
| ZY05719_06525 | -1.404198267 | 5.31E-28 | MazG-like protein |
| ZY05719_00860 | -1.440572591 | 5.83E-05 | hypothetical protein |
| ZY05719_04395 | -1.494764692 | 0.002282788 | asparagine synthetase |
| ZY05719_02195 | -1.584962501 | 0.004996865 | recombination regulator RecX |
| ZY05719_09850 | -1.584962501 | 0.0086413 | 6-phospho-beta-glucosidase |
| ZY05719_07075 | -1.590961241 | 4.61E-19 | PRD domain-containing protein |
| ZY05719_03510 | -1.614709844 | 8.79E-09 | 2%2C6-dichloro-p-hydroquinone 1%2C2-dioxygenase |
| ZY05719_02185 | -1.765534746 | 0.000296414 | tRNA-Pro |
| ZY05719_00665 | -1.94753258 | 0.000549995 | replication initiation factor domain-containing protein |
| ZY05719_04935 | -2.321928095 | 0.003866637 | transporter substrate-binding domain-containing protein |
| ZY05719_03560 | -2.415037499 | 1.55E-05 | thiamine phosphate synthase |
| ZY05719_03735 | -2.584962501 | 0.005885452 | tRNA-Arg |
| ZY05719_07465 | -2.700439718 | 3.26E-05 | winged helix-turn-helix transcriptional regulator |
| ZY05719_00190 | -4.938599455 | 1.46E-22 | rod shape-determining protein MreD |
| ZY05719_02215 | -10.62753388 | 1.15E-234 | GntR family transcriptional regulator |
