# Supplementary material for: Phosphorylation of GntR reduces Streptococcus suis oxidative stress resistance and virulence by inhibiting NADH oxidase transcription
Source: PLoS Pathog. 2023 Mar 13;19(3):e1011227. doi: 10.1371/journal.ppat.1011227 (PMC10010549; doi:10.1371/journal.ppat.1011227)
Supplement: S3 Table — (DOCX) [file ppat.1011227.s012.docx]

**Table S3.** Strains and plasmids used in this study.

| **Name** | **Characteristics** | **Source** |
| --- | --- | --- |
| **Strains** | | |
| ZY05719 | Isolated from a diseased pig in Sichuan, China | Lab stocks |
| Δ*gntR* | Isogenic *gntR* mutant of ZY05719 | This study |
| GntR-S41A | Point-mutation strain of ZY05719 | This study |
| GntR-S41E | Point-mutation strain of ZY05719 | This study |
| GntR-S41E-C*nox* | Restoring *no*x expression in GntR-S41E | This study |
| Δ*nox* | Isogenic *nox* mutant of ZY05719 | This study |
| CΔ*gntR-flag* | Complemented strain of ZY05719 Δ*gntR* with flag | This study |
| NOX^Imp^ | ZY05719 containing *nox* with *impdh* promoter | This study |
| NOX^Eno^ | ZY05719 containing *nox* with *enolase* promoter | This study |
| FDH^Imp^ | ZY05719 containing *fdh* with *impdh* promoter | This study |
| FDH^Eno^ | ZY05719 containing *fdh* with *enolase* promoter | This study |
| *E. coli*DH5α | Cloing host for maintaining the recombinant plasmids | Lab stocks |
| *E. coli* BL21 | The expression host of recombinant proteins | Lab stocks |
| **Plasmids** | | |
| pSET4s | thermosensitive suicide vector; Spc^R^ | Lab stocks |
| pSET2 | *E. coli*–*S. suis* shuttle vector; Spc^R^ | Lab stocks |
| pET28a | Prokaryotic expression | Lab stocks |
| pGEX4T-1 | Prokaryotic expression | Lab stocks |
| pTCV-lacZ | Promoter activity assay | Lab stocks |
| pET28a-*gntR* | Cloning expression of recombinant GntR | This study |
| pET28a-*gntR*-S41A | Cloning expression of recombinant GntR-S41A | This study |
| pET28a-*gntR*-S42A | Cloning expression of recombinant GntR-S42A | This study |
| pET28a-*gntR*-T44A | Cloning expression of recombinant GntR-T44A | This study |
| pET28a-*nox* | Cloning expression of recombinant NOX | This study |
| pGEX4T-1-*nstk* | Cloning expression of recombinant nSTK | This study |
| pSET4s-*gntR* | Recombinant vector designed to knock out *gntR*, Spc^R^ | This study |
| pSET4s-*gntR*-S41A | Recombinant vector designed to point mutation | This study |
| pSET4s-*gntR*-S42A | Recombinant vector designed to point mutation | This study |
| pSET4s-*gntR*-T44A | Recombinant vector designed to point mutation | This study |
| pSET4s-*gntR*-S41E | Recombinant vector designed to point mutation | This study |
| pSET4s-*nox* | Recombinant vector designed to knock out *nox*, Spc^R^ | This study |
| pSET2-*gntR-flag* | pSET2 containing *gntR-flag*, Spc^R^ | This study |
| pSET2-*nox-imp* | pSET2 containing *nox with impdh* promoter, Spc^R^ | This study |
| pSET2-*nox-eno* | pSET2 containing *nox with enolase* promoter, Spc^R^ | This study |
| pSET2-*fdh-imp* | pSET2 containing *fdh with impdh* promoter, Spc^R^ | This study |
| pSET2-*fdh-eno* | pSET2 containing *fdh with enolase* promoter, Spc^R^ | This study |
| pTCV-nox-lacZ | Promoter of *nox* activity assay | This study |
