# Supplementary material for: Phosphorylation of GntR reduces Streptococcus suis oxidative stress resistance and virulence by inhibiting NADH oxidase transcription
Source: PLoS Pathog. 2023 Mar 13;19(3):e1011227. doi: 10.1371/journal.ppat.1011227 (PMC10010549; doi:10.1371/journal.ppat.1011227)
Supplement: S4 Table — (DOCX) [file ppat.1011227.s013.docx]

**Table S4.** Primer used in this study.

| **Primers** | **Sequence(5’-3’)** |
| --- | --- |
| **For strains construction** | |
| Δ*gntR*-F1 | TAAAACGACGGCCAGTGAATTCCAGAGCGTGAGTTGACACAG |
| Δ*gntR*-R1 | TGGAAAGGAAGGAAATAAGTAAAAACGCAGACTAACGTCC |
| Δ*gntR*-F2 | GGACGTTAGTCTGCGTTTTTACTTATTTCCTTCCTTTCCA |
| Δ*gntR*-R2 | GCAGGTCGACTCTAGAGGATCCGTAAACAAGGGAGCAGTTAG |
| GntR-S41A-F1 | CGAGCTCGGTACCCGGGGATCCCTTCATCAACCAAGCAGGGAG |
| GntR-S41A-R1 | CACGGATAACGGTAATGGAGGCAACATTAAATAGTTTTGTTA |
| GntR-S41A-F2 | TAACAAAACTATTTAATGTTGCCTCCATTACCGTTATCCGTG |
| GntR-S41A-R2 | TGACCATGATTACGCCAAGCTTGTCAGACTATATTGCGCAGC |
| GntR-S42A-F1 | Same as GntR-S41A-F1 |
| GntR-S42A-R1 | TGCACGGATAACGGTAATGGCGCTAACATTAAATAGTTTTG |
| GntR-S42A-F2 | CAAAACTATTTAATGTTAGCGCCATTACCGTTATCCGTGCA |
| GntR-S42A-R2 | Same as GntR-S41A-R2 |
| GntR-T44A-F1 | Same as GntR-S41A-F1 |
| GntR-T44A-R1 | GTTTACTGCACGGATAACGGCAATGGAGCTAACATTAAATA |
| GntR-T44A-F2 | TATTTAATGTTAGCTCCATTGCCGTTATCCGTGCAGTAAAC |
| GntR-T44A-R2 | Same as GntR-S41A-R2 |
| GntR-S41E-F1 | Same as GntR-S41A-F1 |
| GntR-S41E-R1 | GCACGGATAACGGTAATGGACTCAACATTAAATAGTTTTGTTA |
| GntR-S41E-F2 | TAACAAAACTATTTAATGTTGAGTCCATTACCGTTATCCGTGC |
| GntR-S41E-R2 | Same as GntR-S41A-R2 |
| Δ*nox*-F1 | CGGAATTCGTCGGAAATAGAATGTCC |
| Δ*nox*-R1 | GAGCTTTTGTATTCAATTAAAATGTAAAATCTCACTTTCG |
| Δ*nox* -F2 | CGAAAGTGAGATTTTACATTTTAATTGAATACAAAAGCTC |
| Δ*nox* -R2 | CGGGATCCCGCTTCAAATGCCTTGTC |
| C△*gntR*-*flag*-F | CGAAGCTTGCTCAATATCCAATATACCC |
| C△*gntR*-*flag*-R | CGGAATTCTTACTTGTCATCGTCGTCCTTGTAGTCGCGATGGTTGGCTGTAATTTC |
| C-*nox*-*imp*-F1 | TGATTACGCCAAGCTTGCATGCATGGAGGCAGGACAGGTAT |
| C-*nox*-*imp*-R1 | ACAACAACGATTTTAGCCATGTTCTTTCCTTTCTTTTGGG |
| C-*nox*-*imp*-F2 | CCCAAAAGAAAGGAAAGAACATGGCTAAAATCGTTGTTGT |
| C-*nox*-*imp*-R2 | CGAGCTCGGTACCCGGGGATCCTTATTCAGCACCCAAGGC |
| C-*nox*-*eno*-F1 | TGATTACGCCAAGCTTGCATGCTGTTTCGCCAGAGGCTTTC |
| C-*nox*-*eno*-R1 | ACAACAACGATTTTAGCCATTATATTACTCTCCTTTGAGT |
| C-*nox*-*eno*-F2 | ACTCAAAGGAGAGTAATATAATGGCTAAAATCGTTGTTGT |
| C-*nox*-*eno*-R2 | Same as C-*nox*-*imp*-R2 |
| C-*fdh*-*imp*-F1 | Same as C-*nox*-*imp*-F1 |
| C-*fdh*-*imp*-R1 | AAATAATGCTACGATTTTCATGTTCTTTCCTTTCTTTTGG |
| C-*fdh*-*imp*-F2 | CCAAAAGAAAGGAAAGAACATGAAAATCGTAGCATTATTT |
| C-*fdh*-*imp*-R2 | CGAGCTCGGTACCCGGGGATCCCTATTTAGCTGTATAACTTTTAC |
| C-*fdh*-*eno*-F1 | Same as C-*nox*-*eno*-F1 |
| C-*fdh*-*eno*-R1 | AATAATGCTACGATTTTCATTATATTACTCTCCTTTGAGT |
| C-*fdh*-*eno*-F2 | ACTCAAAGGAGAGTAATATAATGAAAATCGTAGCATTATT |
| C-*fdh*-*eno*-R2 | Same as C-*fdh*-*imp*-R2 |
| **Prokaryotic expression** | |
| nSTK-F | CGCGGATCCATGATTCAAATCGGTAAGATC |
| nSTK-F | CCGCTCGAGTGTATCAACCTTGTTCCC |
| GntR-F | CGGGATCCATGAAAGTACCGAAGTACCAAC |
| GntR-R | CGGAATTCTTAGCGATGGTTGGCTGTAAT |
| GntR-S41A-R1 (P) | CACGGATAACGGTAATGGAGGCAACATTAAATAGTTTTGTTA |
| GntR-S41A-F2 (P) | TAACAAAACTATTTAATGTTGCCTCCATTACCGTTATCCGTG |
| GntR-S42A-R1 (P) | TGCACGGATAACGGTAATGGCGCTAACATTAAATAGTTTTG |
| GntR-S42A-F2 (P) | CAAAACTATTTAATGTTAGCGCCATTACCGTTATCCGTGCA |
| GntR-T44A-R1 (P) | GTTTACTGCACGGATAACGGCAATGGAGCTAACATTAAATA |
| GntR-T44A-F2 (P) | TATTTAATGTTAGCTCCATTGCCGTTATCCGTGCAGTAAAC |
| GntR-S41E-R1 (P) | GCACGGATAACGGTAATGGACTCAACATTAAATAGTTTTGTTA |
| GntR-S41E-F2 (P) | TAACAAAACTATTTAATGTTGAGTCCATTACCGTTATCCGTGC |
| NOX-F | CGGGATCCATGGCTAAAATCGTTGTTG |
| NOX-R | CGGTCGACTTATTCAGCACCCAAGGC |
| **For RT-qPCR** | |
| *nox*-F(qPCR) | ATCTTGGCTGTTGGTTTC |
| *nox* -R(qPCR) | CCAGTAGCGTTGTCGTAG |
| *gapdh*-F(qPCR) | TAAACTTGACGGTGCTGCAC |
| *gapdh*-R(qPCR) | CCAATTGCTCGCCATCAACT |
| **For promoter activity assay** | |
| Pnox-lacZ-F | CAAATGAATTCCCGGGGATCCGCGTTTTGAACAAGTACG |
| Pnox-lacZ-R | GTATCAACAAGCTGGGGATCCGCCATAATGTAAAATCTC |
| **For EMSA or ChIP** | |
| Promoter-*nox-*F | GCGTTTTGAACAAGTACG |
| Promoter-*nox*-R | GCCATAATGTAAAATCTC |
| Promoter-*gntR-*F | ACTTATTTCCTTCCTTTCC |
| Promoter-*gntR*-R | AATCATCAGGATGGACACG |
| 16S-F | GTAACCTGCCTCATAGCG |
| 16S-R | ATTGCCGAAGATTCCCTA |
